# The correlations of tumor mutational burden among single-region tissue, multi-region tissues and blood in non-small cell lung cancer

**DOI:** 10.1186/s40425-019-0581-5

**Published:** 2019-04-03

**Authors:** Yaxiong Zhang, Lianpeng Chang, Yunpeng Yang, Wenfeng Fang, Yanfang Guan, Aiwei Wu, Shaodong Hong, Huaqiang Zhou, Gang Chen, Xi Chen, Shen Zhao, Qiufan Zheng, Hui Pan, Lanjun Zhang, Hao Long, Haoxian Yang, Xin Wang, Zhesheng Wen, Junye Wang, Hong Yang, Xuefeng Xia, Yuanyuan Zhao, Xue Hou, Yuxiang Ma, Ting Zhou, Zhonghan Zhang, Jianhua Zhan, Yan Huang, Hongyun Zhao, Ningning Zhou, Xin Yi, Li Zhang

**Affiliations:** 1 Department of Medical Oncology, Sun Yat-sen University Cancer Center, State Key Laboratory of Oncology in South China, Collaborative Innovation Center for Cancer Medicine, Guangzhou, China; 2 Geneplus-Beijing Institute, Beijing, China; 3 Department of Thoracic Surgery, Sun Yat-sen University Cancer Center, State Key Laboratory of Oncology in South China, Collaborative Innovation Center for Cancer Medicine, Guangzhou, China; 4 Department of Clinical Research, Sun Yat-sen University Cancer Center, State Key Laboratory of Oncology in South China, Collaborative Innovation Center for Cancer Medicine, Guangzhou, China

**Keywords:** NSCLC, Tissue TMB, tTMB, Blood TMB, bTMB, ITH

## Abstract

High-level tissue tumor mutational burden (tTMB) or blood TMB (bTMB) are associated with better response of immunotherapy in non-small cell lung cancer (NSCLC) patients. However, the correlations of single-region tTMB, multi-region tTMB and bTMB remain to be determined. Moreover, whether intratumor heterogeneity (ITH) has impact on TMB should be clarified. We collected multi-region tumor tissues with matched blood from 32 operative NSCLC and evaluated single-region tTMB, multi-region tTMB and bTMB through a 1021-gene panel sequencing. TMB of > 9 mutations/Mb was classified as high. Besides, we used tTMB fold-change to evaluate the influence of the enrolled region number on tTMB. We found both of single-region tTMB and bTMB showed strong correlations with multi-region tTMB, while the former correlated better (Pearson *r* = 0.94, *P* = 2E-84; Pearson *r* = 0.47, *P* = 0.0067). It showed extremely high specificity (100%) but low sensitivity (43%) when using bTMB to define TMB-high patients, while most false-negative predictions were in early-stage patients. Compared to single region, we found significantly enhanced tTMB fold-change if taking multi-regions for consideration. However, it showed insignificant tTMB fold-change increase if the included regions’ number more than three. Moreover, ITH-high patients had significantly higher tTMB fold-change compared with ITH-low patients (2.32 vs. 1.02, *P* = 8.879e-05). The conversion rate of tTMB level (tTMB-low to tTMB-high) was numerically higher in ITH-high group than that in ITH-low group (16.67% vs. 3.84%). In summary, single-region tTMB has stronger correlation with multi-region tTMB compared with bTMB. ITH has an impact on tTMB, especially in high-level ITH patients.

**Electronic supplementary material:**

The online version of this article (10.1186/s40425-019-0581-5) contains supplementary material, which is available to authorized users.

## Background

Tumor mutational burden (TMB) is emerging as a practical biomarker for predicting the response of immune checkpoint inhibitors (ICIs) [1]. Non-small cell lung cancer (NSCLC) patients with higher tissue TMB (tTMB) or blood TMB (bTMB) levels are associated with better efficacy of ICIs [2, 3]. However, the correlations of single-region tTMB, multi-region tTMB and bTMB remain to be determined. Moreover, whether intratumor heterogeneity (ITH) has impact on TMB should be clarified.

## Methods

We collected multi-region tumor tissues with matched blood from 32 operative NSCLC patients (Additional file 1: Table S1), including epidermal growth factor receptor (EGFR)-mutant lung adenocarcinoma (LUAD) (*n* = 9), kirsten rat sarcoma viral oncogene (KRAS)-mutant LUAD (*n* = 6), EGFR&KRAS-wild-type LUAD (*n* = 11), lung squamous cell carcinoma (LUSC) (*n* = 5) and lymphoepithelioma-like carcinoma (LELC) (*n *= 1). Multi-region tumor tissues (approximate size in 1.5 mm*1.5 mm*1.5 mm for each region at least) were collected in different directions of the primary lesion. Then we explored the correlations among single-region tTMB, multi-region tTMB and bTMB through a 1021-gene panel sequencing (Additional file 1: Table S2). TMB reflected somatic non-synonymous single nucleotide variants (SNVs), insertions, and deletions per megabase of the panel region. Single-region tTMB was defined as the number of somatic non-synonymous mutations per megabase from single region. Multi-region tTMB was calculated with non-repetitive mutations from all regions per megabase. bTMB was analyzed with tumor-derived mutations from ctDNA. TMB of > 9 mutations/Mb was classified as high, using the top quartile threshold of 2000 samples from database of Geneplus. Besides, we used tTMB fold-change, computed by the mean tTMB of multi-regions through a random iterated algorithm divided by that of the single region, to evaluate the influence of the enrolled region numbers on tTMB and explored the impact of ITH on tTMB. ITH is evaluated by ITH index (ITHi). Much more details about methods were shown in Additional file 2: Supplementary Methods.

## Results

We calculated TMB value between our panel sequencing and whole exome sequencing (WES) and observed significant consistency (Additional file 3: Figure S1), indicating that TMB calculated via our panel sequencing is a valid measurement. EGFR-mutant LUAD showed significantly lower multi-region tTMB (median, 4.32, 1.44–14.4) than those in KRAS-mutant LUAD (median, 10.08, 5.76–84.96, *P* = 0.0282) and EGFR&KRAS-wild-type LUAD (median, 14.4, 4.32–38.88, *P* = 0.0281) (Additional file 3: Figure S2).

**Fig. 1:**
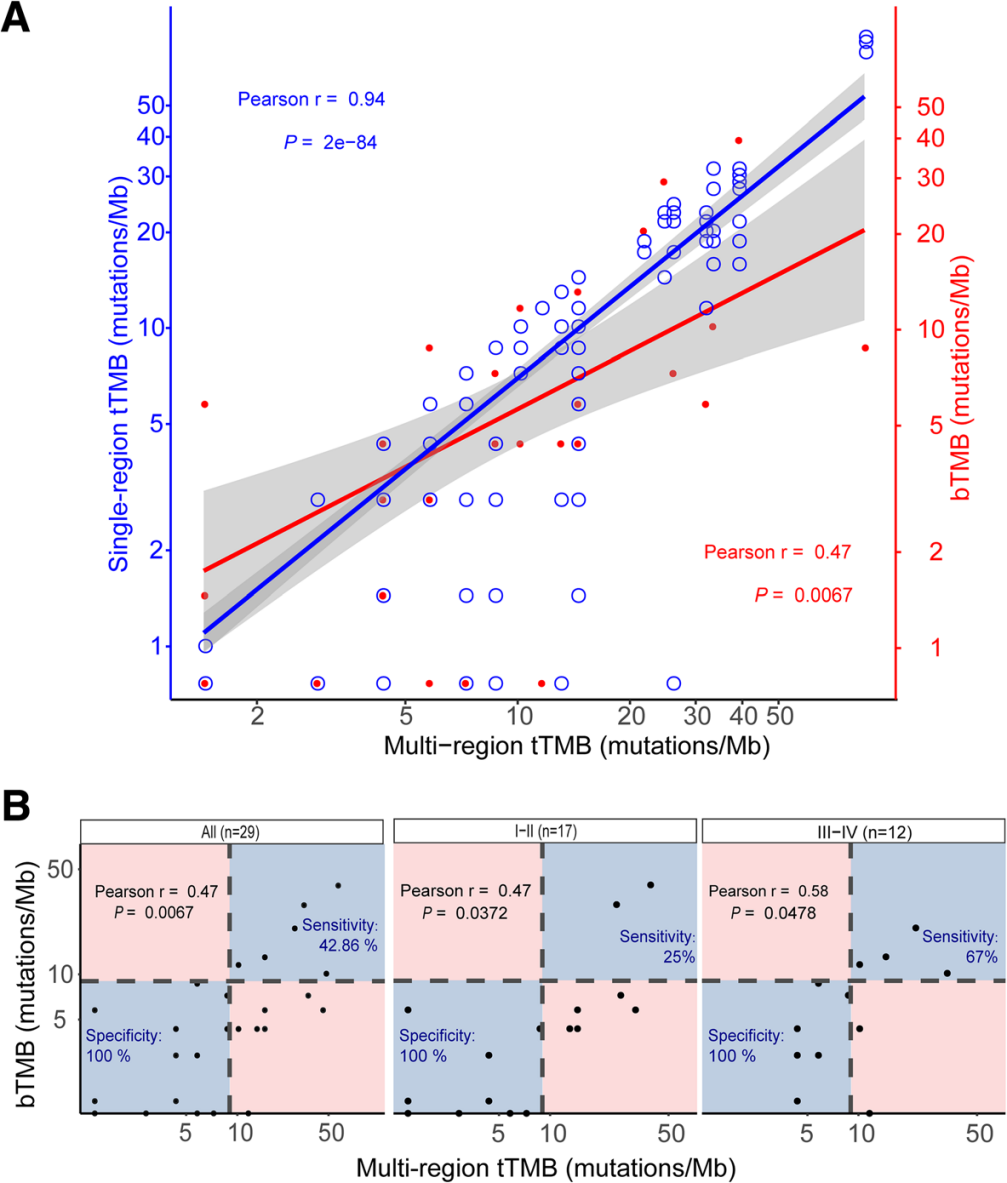
The correlations of TMB among single-region tissue, multi-region tissues and blood. **a** Single-region tTMB vs. multi-region tTMB & bTMB vs. multi-region tTMB. The Pearson correlations were performed between single-region tTMB and multi-region tTMB (blue, *r* = 0.94), and between bTMB and multi-region tTMB (red, *r* = 0.47). TMB reflected somatic non-synonymous SNVs, insertions, and deletions per megabase of the panel region. Single-region tTMB was defined as the number of somatic non-synonymous mutations per megabase from single region. Multi-region tTMB was calculated with non-repetitive mutations from all regions per megabase. bTMB was analyzed with tumor-derived mutations from ctDNA. **b** bTMB level evaluation using multi-region tTMB level as standard. The Pearson correlations between bTMB and multi-region tTMB in all patients (left), I-II stage patients (middle), and III-IV stage patients (right) were 0.47, 0.47 and 0.58, respectively. Tumor stage were evaluated following the guidelines in the International Staging System for Lung Cancer, 7th edition. The sensitivity (true positive rate: the number of high-level bTMB & high-level multi-region tTMB patients divided by the number of high-level multi-region tTMB patients) and specificity (true negative rate: the number of low-level bTMB & low-level multi-region tTMB patients divided by the number of low-level multi-region tTMB patients) were analysed using multi-region tTMB level as golden standard. TMB of > 9 mutations/Mb was classified as high, using the top quartile threshold of 2000 samples from database of Geneplus. Abbreviations: TMB, tumor mutational burden; tTMB, tissue TMB; bTMB, blood TMB; *r*, Pearson correlation coefficient

Both of single-region tTMB and bTMB showed strong correlations with multi-region tTMB, while the former correlated better (Pearson *r* = 0.94, *P* = 2E-84, Pearson *r* = 0.47, *P* = 0.0067) (Fig. 1a). It showed extremely high specificity (100%) but relatively low sensitivity (43%) when using bTMB to define TMB-high patients, while most false-negative predictions were in early-stage (I-II) patients (Fig. 1b). The classification accuracy was higher in late-stage (III-IV) patients (83%) than that in early-stage (I-II) patients (70%). Additional file 3: Figure S3 made the comparisons and overlaps of tumor-derived mutational profiles among each region of tumor tissues and the corresponding ctDNA for each enrolled patient. It exhibited extensive heterogeneity of the mutational profile overlaps between ctDNA and the tumor DNA.

**Fig. 2:**
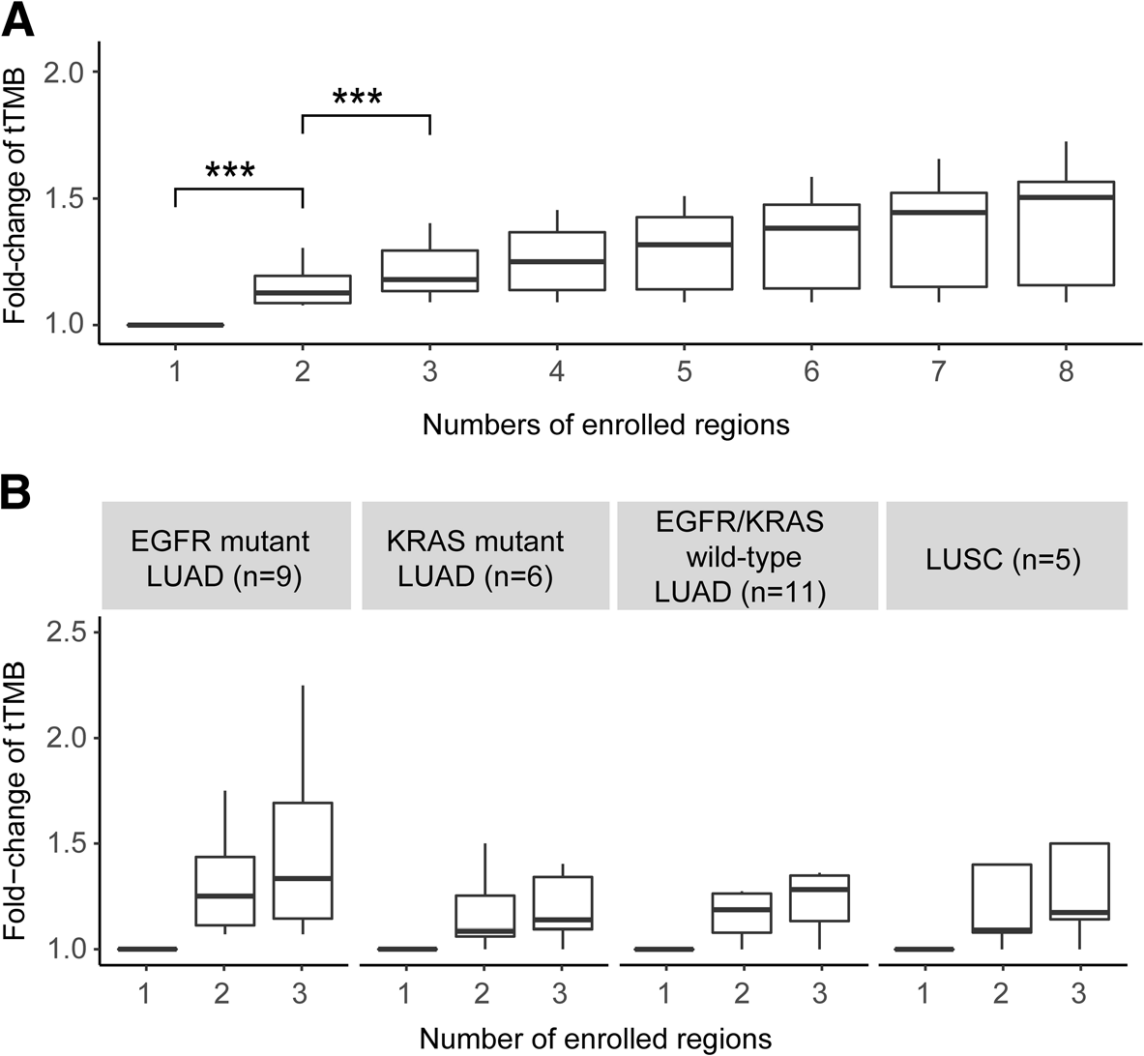
tTMB fold-change based on multi-regions compared with single region. **a** For overall patients. For overall analysis, tTMB fold-change is computed by the mean tTMB of two-regions, three-regions, four-regions, five-regions, six-regions, seven-regions or eight-regions through a random iterated algorithm, then divided by that of the single region. **b** For different NSCLC subtypes. For subgroup analysis, tTMB fold-change is computed by the mean tTMB of two-regions or three-regions through a random iterated algorithm, then divided by that of the single region, because it shows insignificant tTMB fold-change increase if the included regions’ number more than three. Abbreviations: TMB, tumor mutational burden; tTMB, tissue TMB

Compared to single region, we found significantly enhanced tTMB fold-change if taking multi-regions for consideration. However, it showed insignificant tTMB fold-change increase if the included regions’ number more than three (Fig. 2a). Subgroup analysis exhibited similar trends among different molecular subtypes of NSCLC, especially in EGFR-mutant LUAD (Fig. 2b).

**Fig. 3:**
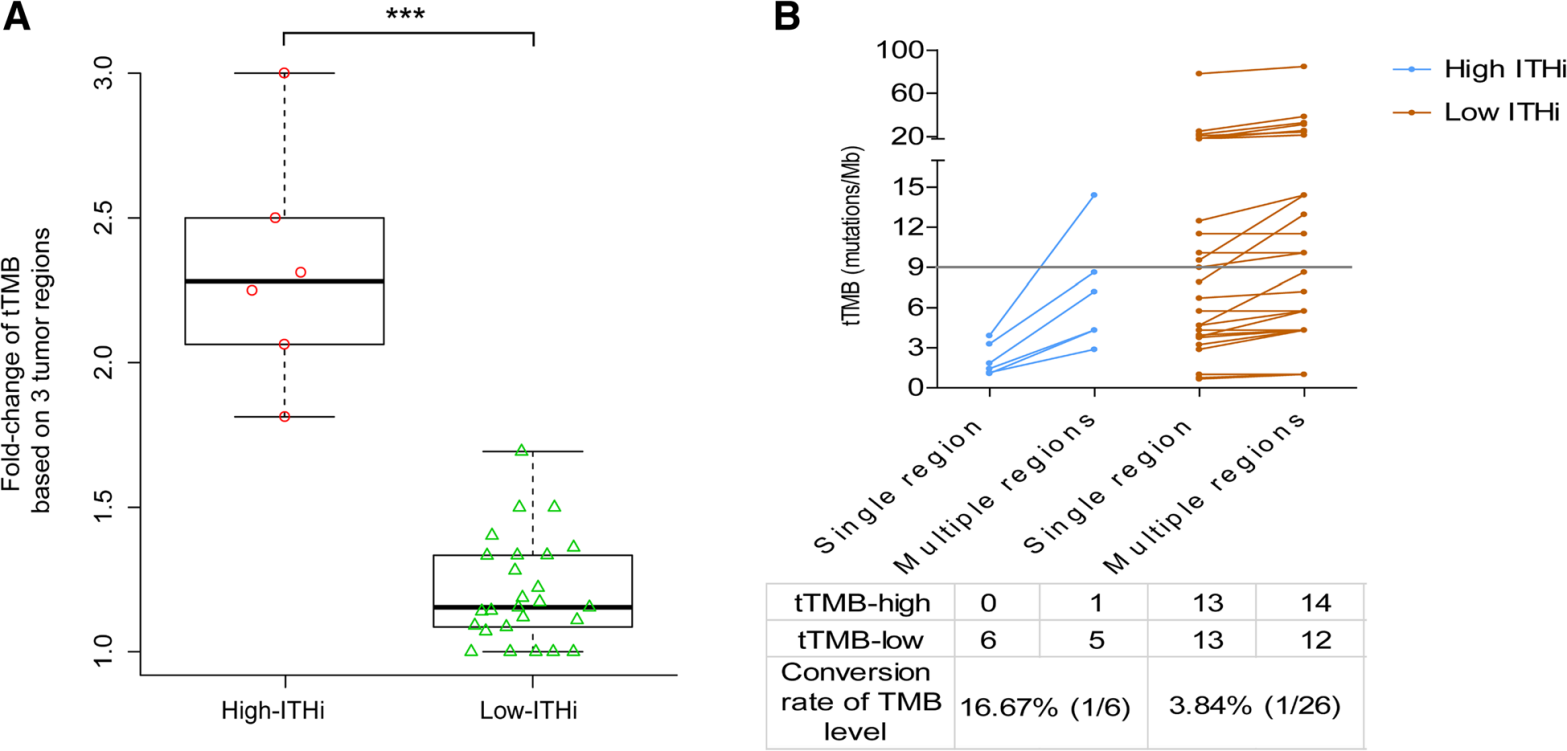
Impact of ITH on TMB assessment. **a** tTMB fold-change comparison between ITHi-high and ITHi-low patients. tTMB fold-change is computed by the mean tTMB of three-regions through a random iterated algorithm, then divided by that of the single region. **b** The conversion rate of tTMB level status between ITHi-high and ITHi-low patients if taking multi-region tTMB analysis. ITH index (ITHi) was calculated with specific formula displayed in Additional file 2: Supplementary Methods. ITHi ranges from 0 (lowest ITH) to 1 (highest ITH). If the tumor has less shared somatic genetic alterations after multi-region sequencing, the ITHi of this tumor will be higher. Otherwise, the ITHi of this tumor will be lower. Abbreviations: ITH, intratumor heterogeneity; TMB, tumor mutational burden; tTMB, tissue TMB

ITH-high patients had significantly higher tTMB fold-change compared with ITH-low patients (2.32 vs. 1.02, *P* = 8.879e-05) (Fig. 3a). The conversion rate of tTMB level status (tTMB-low to tTMB-high) in the ITH-high group was numerically higher than that in the ITH-low group (16.67% vs. 3.84%) if taking multi-region tTMB analysis (Fig. 3b).

## Discussion

We found single-region tTMB had stronger correlation with multi-region tTMB compared with bTMB, thus revealed the limitation of ctDNA for TMB analysis. It showed low sensitivity of bTMB in TMB level evaluation using multi-region tTMB level as standard,while most false-negative predictions existed in early-stage patients. It may be due to much lower circulating tumor DNA (ctDNA) released into blood in early-stage NSCLC, which involves a smaller tumor load and/or less metastasis [4]. Although bTMB might not correlate as well with tTMB as much as the single and multi-region tTMB measurements between themselves, bTMB could still be predictive for ICI response in advanced NSCLC [3, 5]. One possible reason is not just the number of mutations per se that is important, but the quality of these mutations and the number and quality of immune-actionable neo-antigens they generate. Thus, bTMB may be predictive if it detects the “useful” or “immune actionable” mutations as well as tTMB. This is the real issue in this field and why simple measures of overall TMB are really emerging to be useless as a biomarker. More prospective trials are still necessary to validate the reliability, stability and the cut-off value of bTMB as a biomarker for ICI efficacy. We also need to explore the real immune actionable mutational profiles that may be a better biomarker for ICI efficacy compared with overall TMB.

Moreover, we found ITH had an impact on tTMB estimation, especially in high-level ITH patients. Surprisingly, after considering more sampling regions, some patients who were considered low-TMB previously, were reclassified as high-TMB. A recent study demonstrated clinical benefit from ICI therapy for NSCLC patients with high-TMB [6]. However, another study showed a part of (18.2 to 35.3%) low-TMB NSCLC patients still had benefit from ICIs [2]. Our study revealed that single region sampling would underestimate the tTMB level in patients with high ITH, perhaps providing an explanation for why some low-TMB patients evaluated by a single region biopsy still achieve benefit from ICIs. Use of multi-region sampling methods may consequently result in more comprehensive and accurate tTMB evaluation, allowing identification of high-TMB patients, especially those with high ITHi, such as EGFR-mutant LUAD, as these patients may reap benefit from ICIs.

Major limitations of this study were the sample size and the single-centered design. Besides, all of the enrolled patients received no ICIs, so we could not evaluated therapeutic efficacy based on single-region tTMB, multi-region tTMB and bTMB in post hoc analysis.

## Conclusions

Single-region tTMB has stronger correlation with multi-region tTMB compared with bTMB, revealing potential limitation of TMB analysis using ctDNA. ITH has an impact on tTMB, especially in high-level ITH patients, thus firstly demonstrating tTMB heterogeneity and providing an explanation for why some low-TMB patients evaluated by a single region biopsy still achieve benefit from ICIs.

## Additional files

Additional file 1: **Table S1.** Clinical characteristics of the enrolled 32 NSCLC patients. **Table S2.** List of target regions of the pan-cancer 1021-gene panel. (DOCX 43 kb)

Additional file 2: Supplementary Methods. (DOCX 24 kb)

Additional file 3 **Figure S1.** The correlations of TMB value between panel sequencing and WES in published databases. **Figure S2.** The comparison of multi-region tTMB among different NSCLC subtypes. **Figure S3.** The comparisons and overlaps of tumor-derived mutational profiles among tumor tissues in each region and the corresponding ctDNA. (PDF 1439 kb)
