# Supplementary material for: The correlations of tumor mutational burden among single-region tissue, multi-region tissues and blood in non-small cell lung cancer
Source: J Immunother Cancer. 2019 Apr 3;7:98. doi: 10.1186/s40425-019-0581-5 (PMC6448263; doi:10.1186/s40425-019-0581-5)
Supplement: Supplementary file 1 — Table S1. Clinical characteristics of the enrolled 32 NSCLC patients. Table S2. List of target regions of the pan-cancer 1021-gene panel. (DOCX 43 kb) [file 40425_2019_581_MOESM1_ESM.docx]

**Table S1. Clinical characteristics of the enrolled 32 NSCLC patients.**

| **Clinical characteristics** | | **Patients(n)** |
| --- | --- | --- |
| **Age: median(range)**  57(45-79) | <60 | 18 |
|  | ≥60 | 14 |
| **Gender** | Male | 25 |
|  | Female | 7 |
| **NSCLC subtypes** | EGFR mutant LUAD | 9 |
|  | KRAS mutant LUAD | 6 |
|  | EGFR&KRAS-wild-type LUAD | 11 |
|  | LUSC | 5 |
|  | LELC | 1 |
| **TNM stage^*^** | Ia | 4 |
|  | Ib | 8 |
|  | IIa | 3 |
|  | IIb | 5 |
|  | IIIa | 8 |
|  | IIIb | 2 |
|  | IV^#^ | 2 |
| **Lymph node metastasis** | Yes | 15 |
|  | No | 17 |
| **Drinking status** | drinker | 16 |
|  | non-drinker | 16 |
| **Smoking status** | smoker | 21 |
|  | non-smoker | 11 |

* AJCC Version 7

# Both patients had solitary brain metastasis and received brain radical surgery treatment.

Abbreviations: NSCLC: non-small cell lung cancer; EGFR: epidermal growth factor receptor; KRAS: kirsten rat sarcoma viral oncogene; LUAD: lung adenocarcinoma; LUSC: lung squamous cell carcinoma; LELC: lymphoepithelioma-like carcinoma.

**Table S2. List of target regions of the pan-cancer 1021-gene panel.**

| **Coding sequence** | | | | | | | | |
| --- | --- | --- | --- | --- | --- | --- | --- | --- |
| ABL1 | BRD3 | CDKN2B | FAT1 | HDAC1 | MCL1 | NOTCH3 | PTEN | SYK |
| ABL2 | BRD4 | CHEK1 | FBXW7 | HDAC4 | MDM2 | NOTCH4 | PTPN11 | TMPRSS2 |
| AKT1 | BTK | CHEK2 | FCGR2A | HGF | MDM4 | NRAS | RAF1 | TOP1 |
| AKT2 | C11orf30 | CRKL | FCGR2B | HRAS | MED12 | NTRK1 | RARA | TP53 |
| AKT3 | C1QA | CSF1R | FCGR3A | IDH1 | MET | NTRK3 | RB1 | TSC1 |
| ALK | C1S | CTNNB1 | FGFR1 | IDH2 | MITF | PALB2 | RET | TSC2 |
| APC | CBL | DDR1 | FGFR2 | IGF1R | MLH1 | PDGFRA | RHEB | VEGFA |
| AR | CCND1 | DDR2 | FGFR3 | IL7R | MLH3 | PDGFRB | RHOA | VHL |
| ARAF | CCND2 | DNMT3A | FGFR4 | INPP4B | MPL | PDK1 | RICTOR | XPO1 |
| ATM | CCND3 | EGFR | FLCN | IRS2 | MS4A1 | PIK3CA | RNF43 | XRCC1 |
| ATR | CCNE1 | EPHA2 | FLT1 | JAK1 | MSH2 | PIK3CB | ROCK1 |  |
| AURKA | CD274 | EPHA3 | FLT3 | JAK2 | MSH3 | PIK3R1 | ROS1 |  |
| AURKB | CDH1 | EPHA5 | FLT4 | JAK3 | MSH6 | PIK3R2 | RPS6KB1 |  |
| AXL | CDK13 | ERBB2 | FOXA1 | KDR | MTOR | PMS1 | SMARCA4 |  |
| BAP1 | CDK4 | ERBB3 | FOXL2 | KIT | MYC | PMS2 | SMARCB1 |  |
| BCL2 | CDK6 | ERBB4 | GAB2 | KRAS | MYD88 | PRKAA1 | SMO |  |
| BRAF | CDK8 | ERCC1 | GATA3 | MAP2K1 | NF1 | PSMB1 | SRC |  |
| BRCA1 | CDKN1A | ERG | GNA11 | MAP2K2 | NF2 | PSMB5 | STAT1 |  |
| BRCA2 | CDKN1B | ESR1 | GNAQ | MAPK1 | NOTCH1 | PTCH1 | STAT3 |  |
| BRD2 | CDKN2A | EZH2 | GNAS | MAPK3 | NOTCH2 | PTCH2 | STK11 |  |
| **Hot exons** | | | | | | | | |
| ABCA10 | CAPRIN1 | DMXL1 | GLYR1 | LMAN1L | NXF5 | RALBP1 | STAG2 | UNC13A |
| ABCA8 | CARS | DMXL2 | GMDS | LMBR1L | OBP2A | RAPGEF2 | STAT4 | UNC13D |
| ABCB7 | CARS2 | DNAH10 | GNPTAB | LPCAT4 | OBP2B | RARB | STAT6 | UNC5D |
| ABCC8 | CASC4 | DNAH5 | GOLGA4 | LPHN3 | OCA2 | RASEF | STK11IP | USP12 |
| ABCF2 | CASP8 | DNAH9 | GPAT2 | LRBA | ODZ3 | RBM6 | STK31 | USP34 |
| ACE | CASP8AP2 | DNAJC11 | GPATCH2 | LRP1B | OR2T4 | RBMX | STX3 | USP39 |
| ACER2 | CASQ2 | DNAJC9 | GPR114 | LRP2 | OR4A15 | RCC1 | SULT1A4 | USP45 |
| ACOT11 | CATSPER2 | DNTTIP1 | GPR125 | LRP4 | OR4C6 | REC8 | SUPT5H | USP48 |
| ACPP | CBFB | DOCK11 | GPR133 | LRRC16B | OR5L2 | REG1B | SUPT6H | VAV1 |
| ACSL1 | CBX4 | DOCK3 | GPR144 | LRRC2 | OR6F1 | RELN | SYCP2L | VEZF1 |
| ACSM5 | CCDC155 | DOT1L | GPS2 | LRRC7 | OSBPL10 | RERE | SYNE1 | VILL |
| ACSS3 | CCDC159 | DPP10 | GRIA3 | LRRC72 | OTOA | RFWD2 | SYNE2 | VIT |
| ACTL6B | CCDC17 | DPP4 | GRIK2 | LRRD1 | OTOGL | RFX3 | SYNJ2 | VPS13A |
| ADAM23 | CCT3 | DRGX | GUCY1A3 | LRRFIP2 | OVCH1 | RNF215 | TAF1B | VPS33B |
| ADAM33 | CCT6B | DUOX1 | GUCY2C | LRSAM1 | P4HB | RNF219 | TAF6 | VSIG4 |
| ADAMTS12 | CD1E | DYSF | GYLTL1B | LTBP1 | PABPC4 | RPL22 | TARBP1 | WAS |
| ADAMTS16 | CD300LF | DZANK1 | HAAO | LUC7L2 | PACS2 | RPL36A | TBC1D1 | WASL |
| ADAMTS19 | CD5L | ECHDC1 | HAP1 | LUZP4 | PAEP | RPS5 | TBC1D21 | WDR44 |
| ADAMTS20 | CD9 | EDN1 | HAUS5 | MAEL | PAGE1 | RPS6KA1 | TBC1D3 | WDR52 |
| ADAMTS5 | CD97 | EEF1A1 | HAUS6 | MAGI1 | PARK2 | RPTOR | TBC1D5 | WDR62 |
| ADAMTSL1 | CD99 | EFCAB5 | HCN1 | MAN2A1 | PARP4 | RPUSD4 | TBL1X | WDR66 |
| ADD2 | CDH18 | EFCAB6 | HDAC6 | MAP2 | PCK2 | RREB1 | TBP | WDR72 |
| AGMAT | CDH24 | EFCAB7 | HEATR7B2 | MAP2K4 | PCLO | RRP7A | TBX15 | WDTC1 |
| AGTPBP1 | CDH26 | EFHA2 | HECTD4 | MAP3K1 | PCNT | RUNDC3A | TBX22 | WLS |
| AHCTF1 | CDK11A | EFNA5 | HECW1 | MAP4K1 | PCNXL2 | RUNX1 | TBX3 | WSCD2 |
| AK5 | CDK12 | EIF1AX | HECW2 | MAPKAPK3 | PCSK5 | RYR2 | TCF20 | WWP2 |
| AKR1B10 | CDK14 | EIF2B5 | HID1 | MAPRE3 | PCYT1A | RYR3 | TCF4 | XBP1 |
| AKR1C1 | CDK18 | EIF2C2 | HIST1H3B | MAST1 | PDCD6 | SAFB2 | TCP10 | XPO4 |
| ALDH1A3 | CDK19 | EIF3E | HLA-DRB1 | MBIP | PDE1C | SAG | TCP11 | XPO5 |
| ALDH2 | CDS1 | EIF3I | HLA-DRB5 | MBTPS2 | PDE2A | SAGE1 | TEK | ZAP70 |
| ALG5 | CEACAM20 | EIF4ENIF1 | HMCN1 | MCF2L2 | PDE4DIP | SAMD8 | TERT | ZBTB8OS |
| ALX4 | CECR2 | EIF4H | HMHA1 | MCOLN2 | PDIA5 | SCN10A | TESC | ZC3H13 |
| AMOT | CELA2B | ELAVL3 | HNF4A | MDGA2 | PDILT | SCN3A | TEX35 | ZC3H7B |
| ANK2 | CGN | ELL3 | HOMER2 | MDN1 | PDRG1 | SCN7A | TFDP1 | ZDHHC11 |
| ANKRD13D | CHD3 | EMID2 | HPS3 | MED23 | PEX6 | SCN9A | TGDS | ZFC3H1 |
| ANKRD20A4 | CHD4 | ENPP2 | HPS4 | MEFV | PGAP1 | SDK2 | TGM2 | ZFR |
| ANKRD27 | CHD6 | ENTPD6 | HSPA12B | METTL14 | PHACTR3 | SEC14L4 | TGM5 | ZMYM4 |
| ANKRD28 | CHI3L1 | EPB41L2 | HSPD1 | METTL5 | PHF20L1 | SEC24B | THBS2 | ZNF143 |
| ANKRD30A | CISD3 | EPB41L4B | HYDIN | MGAM | PHYH | SEH1L | THEM5 | ZNF350 |
| ANKRD30B | CLCN7 | EPHB1 | IBSP | MICALL1 | PI4KB | SELP | THOC1 | ZNF385A |
| ANKRD36B | CLEC16A | EPS8L3 | IFT172 | MID1 | PIP4K2C | SEMA6A | THSD7A | ZNF414 |
| ANO2 | CLINT1 | ESD | IGSF9 | MIER2 | PIP5K1C | SEPT12. | THSD7B | ZNF512B |
| AP1B1 | CNGB3 | ETNK2 | IKBKAP | MLL3 | PIWIL1 | SERPINA7 | TIMD4 | ZNF541 |
| AP1G2 | CNKSR2 | ETV6 | IKBKE | MLPH | PKD1L2 | SETD1B | TIMM44 | ZNF563 |
| AP3B1 | CNOT3 | EXOC4 | IL11RA | MORC1 | PKHD1 | SETD2 | TIMP3 | ZNF614 |
| APAF1 | CNOT4 | EXOC5 | IL13RA2 | MORN1 | PKLR | SF1 | TJP3 | ZNF687 |
| APLP2 | CNTN1 | EXOC6 | IL1RAPL1 | MRPL1 | PLAC8 | SF3B1 | TLE1 | ZNF705B |
| APMAP | CNTN4 | EXOC7 | IL27RA | MRPL24 | PLCB4 | SF3B14 | TLL1 | ZNF705G |
| APPL2 | CNTN5 | EXTL3 | IMPG1 | MRPS18B | PLCZ1 | SF3B3 | TMC2 | ZNF711 |
| AQP12A | CNTNAP3B | EYA4 | INHBA | MSI1 | PLEC | SGCZ | TMED8 | ZNF804B |
| ARFGAP1 | CNTNAP5 | F8 | INPP5J | MTA2 | PLK2 | SGIP1 | TMEM104 | ZSWIM8 |
| ARFRP1 | COASY | F9 | IQCA1 | MTM1 | PLOD3 | SGK1 | TMEM120B |  |
| ARHGAP35 | COL14A1 | FAH | ITFG2 | MTR | PLXNA1 | SGPL1 | TMEM132D |  |
| ARHGAP40 | COL16A1 | FAM114A2 | ITGA8 | MTTP | POLDIP2 | SH2D3A | TMEM145 |  |
| ARHGEF1 | COL19A1 | FAM131B | ITGA9 | MUC5B | POLE | SH3BGR | TMEM247 |  |
| ARHGEF7 | COL1A1 | FAM135B | ITIH1 | MUS81 | POLR2J | SH3PXD2A | TMEM80 |  |
| ARNTL | COL25A1 | FAM13C | ITLN2 | MYB | POLR3B | SHISA4 | TMEM87A |  |
| ARPC4-TTLL3 | COL4A5 | FAM157B | ITM2A | MYBPC2 | POLR3GL | SI | TMTC4 |  |
| ASH2L | COL4A6 | FAM177B | ITPKB | MYCBP2 | POLRMT | SIDT2 | TMX3 |  |
| ASTN1 | COL5A1 | FAM21A | ITPR1 | MYH15 | POM121L12 | SIK3 | TNFAIP6 |  |
| ASXL2 | COL5A2 | FAM3A | KCNAB2 | MYH2 | POTEG | SIM1 | TNFSF4 |  |
| ATAD2B | COL5A3 | FAM49A | KCNH6 | MYH4 | PPA1 | SIM2 | TNN |  |
| ATG9B | COL6A5 | FAM49B | KCNQ2 | MYH8 | PPDPF | SLC13A3 | TNNT1 |  |
| ATP10B | COL6A6 | FAM5C | KDM4A | MYH9 | PPEF1 | SLC17A6 | TNR |  |
| ATP10D | COL9A1 | FAM86B1 | KDM6A | MYL5 | PPFIBP2 | SLC17A8 | TNS3 |  |
| ATP12A | COPA | FAN1 | KEAP1 | MYL6 | PPIL2 | SLC25A1 | TP53BP1 |  |
| ATP2C1 | COPG1 | FANCC | KIAA0195 | MYLK2 | PPP1R17 | SLC25A30 | TPCN1 |  |
| ATP6V0A2 | CPA1 | FASTK | KIAA0226 | MYO3A | PPP4R4 | SLC26A3 | TPH2 |  |
| ATP8B2 | CPSF3 | FATE1 | KIAA0319 | MYOM1 | PQBP1 | SLC2A2 | TPMT |  |
| ATXN2 | CPSF6 | FBN2 | KIAA0922 | NACAD | PREB | SLC30A5 | TPTE |  |
| ATXN7L2 | CRTAM | FDCSP | KIAA1191 | NARF | PREX2 | SLC35B2 | TRIM33 |  |
| BAX | CRTAP | FLNC | KIAA1199 | NAT10 | PRKACA | SLC35B4 | TRIM51 |  |
| BBS9 | CRYBG3 | FLOT2 | KIAA1211L | NAV3 | PRKAG3 | SLC38A4 | TRIM58 |  |
| BCAS1 | CSMD1 | FLT3LG | KIF13A | NBPF1 | PRKCD | SLC38A5 | TRIML1 |  |
| BCAS2 | CSMD3 | FMN2 | KIF1B | NBPF10 | PRKDC | SLC43A1 | TRIO |  |
| BCL2L11 | CSN3 | FMNL3 | KIF26B | NCF2 | PRKX | SLC45A1 | TRIP11 |  |
| BCR | CSNK1E | FNDC4 | KIF5B | NCKAP1 | PRRX1 | SLC4A10 | TRMT112 |  |
| BLOC1S1 | CSPP1 | FNIP2 | KIFAP3 | NCOR1 | PRSS1 | SLC4A4 | TRPC5 |  |
| BMPR1B | CTCF | FOLH1 | KIFC1 | NCOR2 | PRUNE | SLC5A1 | TRUB1 |  |
| BRF1 | CTIF | FOXJ2 | KIR2DL3 | NEK5 | PSG2 | SLC6A5 | TSGA10 |  |
| BRSK2 | CTNNA2 | FRG1 | KIR3DL3 | NELL1 | PSG5 | SLC8A1 | TSKS |  |
| BRWD3 | CTSF | FRG2B | KLHL1 | NFE2L2 | PSIP1 | SLCO1B7 | TSPAN12 |  |
| BSG | CYP2A13 | FRMD4A | KLHL14 | NIPBL | PSMC4 | SLCO5A1 | TSR2 |  |
| BTNL3 | CYP3A4 | FRMPD2 | KLK1 | NLGN3 | PSMC6 | SMTN | TTF2 |  |
| BTRC | CYP4A11 | FRMPD4 | KMT2B | NLRC3 | PSTPIP1 | SNTG1 | TTN |  |
| C12orf5 | CYTH4 | FSD2 | KMT2C | NLRP4 | PTBP3 | SORCS3 | TUBA3C |  |
| C19orf38 | DCLK2 | FSHR | KRT2 | NMI | PTCD3 | SPAG16 | TUBGCP4 |  |
| C1orf112 | DCST1 | FUBP1 | KRT9 | NOP2 | PTGES3L-AARSD1 | SPATA13 | TUBGCP5 |  |
| C1orf35 | DDB1 | FUNDC1 | KRTAP5-5 | NOS1 | PTGS2 | SPG20 | TYK2 |  |
| C20orf112 | DDX24 | GAB3 | KTN1 | NOS2 | PTPLAD1 | SPINT1 | TYRP1 |  |
| C2orf47 | DDX3X | GABRD | L3MBTL1 | NRXN1 | PTPN13 | SPPL2A | U2AF1 |  |
| C2orf62 | DEPDC4 | GAD2 | LARP1 | NRXN2 | PTPRA | SPPL3 | U2AF2 |  |
| C7orf53 | DGKK | GALNT13 | LCN10 | NT5C3L | PTPRD | SPRED1 | UBASH3A |  |
| C9orf114 | DHCR24 | GALNT14 | LCT | NTM | PTPRM | SPTA1 | UBE2Q1 |  |
| C9orf43 | DHDDS | GFRAL | LCTL | NUDCD2 | PYHIN1 | SRRT | UBE4B |  |
| CACNA1A | DHX9 | GIGYF1 | LETM1 | NUP205 | QRICH2 | SSBP3 | UCHL3 |  |
| CACNA1D | DIAPH1 | GINS4 | LGALS13 | NUP210 | RAB1B | SSH2 | UCK2 |  |
| CACNA1E | DKC1 | GIPR | LILRB3 | NUTM1 | RAB3GAP2 | SSPO | UGT8 |  |
| CADM2 | DLST | GKN2 | LILRB4 | NWD1 | RAB6A | ST18 | ULK3 |  |
| CAMKK1 | DMD | GLB1L3 | LIPN | NXF1 | RAC2 | ST6GALNAC1 | UMOD |  |
