# Supplementary material for: The correlations of tumor mutational burden among single-region tissue, multi-region tissues and blood in non-small cell lung cancer
Source: J Immunother Cancer. 2019 Apr 3;7:98. doi: 10.1186/s40425-019-0581-5 (PMC6448263; doi:10.1186/s40425-019-0581-5)
Supplement: Supplementary file 2 — Supplementary Methods. (DOCX 24 kb) [file 40425_2019_581_MOESM2_ESM.docx]

**Supplementary Methods**

**Study populations and ethics approval**

All enrolled non-small cell lung cancer (NSCLC) patients received pulmonary surgery at the Sun Yat-Sen University Cancer Center from June 2016 to March 2017. We collected multi-region tumor tissues with matched peripheral blood and clinical information from each patient. All patients provided written informed consent. This study was approved by the Institutional Review Board (IRB) of Sun Yat-Sen University Cancer Center (IRB number B2017-067-01).

**Targeted capture sequencing**

Peripheral blood was collected in EDTA Vacutainer tubes (BD Diagnostics, Franklin Lakes, NJ, USA) and processed within 3 h. Plasma was separated by centrifugation at 2,500 ×g for 10 min, transferred to microcentrifuge tubes, and then centrifuged at 16,000×g for 10 min to remove remaining cell debris. Peripheral blood lymphocytes (PBLs) from the first centrifugation step were used for the extraction of germline genomic DNA (gDNA). The gDNA of PBL and tissue samples were extracted using the DNeasy Blood & Tissue Kit (Qiagen, Hilden, Germany).To detect ctDNA, circulating cell-free DNA (cfDNA) was isolated from 0.6–1.8 mL plasma using QIAamp Circulating Nucleic Acid Kit (Qiagen). DNA concentration was measured using a Qubit fluorometer (Invitrogen, Carlsbad, CA, USA) and the Qubit dsDNA HS (High Sensitivity) Assay Kit (Invitrogen). The size distribution of the cfDNA was assessed using an Agilent 2100 BioAnalyzer and the DNA HS kit (Agilent Technologies, Santa Clara, CA, USA). All DNA extractions were performed according to the manufacturer’s instructions. Sequencing libraries of both cfDNA and gDNA were constructed with the KAPA DNA Library Preparation Kit (Kapa Biosystems, Wilmington, MA, USA) according to the manufacturer’s protocol. Libraries were hybridized to custom-designed biotinylated oligonucleotide probes (Integrated DNA Technologies, Iowa, IA, USA). Capture probe was designed to cover coding sequencing or hot exons of 1,021 genes frequently mutated in solid tumors.DNA sequencing was performed using the HiSeq 3000 Sequencing System (Illumina, San Diego, CA, USA) with 2×101-bp paired-end reads.

Targeted capture sequencing revealed a mean effective depth of coverage of 816 ×in multi-region tissue samples and 1901 × in ctDNA samples. Single nucleotide variants (SNVs) were called using MuTect(version 1.1.4) and NChot, a software developed in-house to review hotspot variants. Small insertions and deletions (Indels) were called by GATK. Somatic copy-number alterations were identified with CONTRA (v2.0.8). Copy number variations (CNV) was expressed as the ratio of adjusted depth between ct DNA and germline DNA. Mutations were considered a candidate somatic mutation only when (i) the mutation was detected in at least 5 high-quality reads containing the particular base,(ii) the mutation was not present in >1% of the population in the 1000 Genomes Project (version phase 3) or dbSNP databases (The Single Nucleotide Polymorphism Database, version dbSNP 137), and (iii) the mutation was not present in a local database of normal samples. High-quality reads were selected with Phred score ≥30, mapping quality≥30, and a lack of paired-end reads bias. For tumor somatic mutations, the mutant allele must be present in ≥1% of reads. The candidate variants were all manually verified in the Integrative Genomics Viewer.

**TMB analysis**

TMB reflected somatic non-synonymous SNVs, insertions, and deletions per megabase of the panel region. One-region tTMB was defined as the number of somatic non-synonymous mutations per megabase from single region. Multi-region tTMB was calculated with non-repetitive mutations from all regions per megabase. bTMB was analyzed with tumor-derived mutations from ctDNA. TMB of >9 mutations/Mb was classified as high, using the top quartile threshold of 2000 samples from database of Geneplus. Besides, we used tTMB fold-change, computed by the mean tTMB of multi-regions through a random iterated algorithm divided by that of the single region, to evaluate the influence of the enrolled region numbers on tTMB and explored the impact of ITH on tTMB.

**Intratumor heterogeneity (ITH) evaluation**

To eliminate bias introduced by the number of tumor regions, ITH index (ITHi) was evaluated for each patient based on the presence of each detected genetic variation (SNV, indel, gene fusion, and CNV) in tumor regions with more than one variation and expressed as the mean Jaccard distance between variation sets of each of two regions. Patients were excluded from ITHi analysis when (i) only one region contained more than one mutation or (ii) only one mutation was detected in all regions.

$$\begin{aligned} ITH index=\frac{1}{C_{\left| R \right|}^{2}}\sum_{1\leq i<j\leq\left| R \right|} d_{J}\left( V_{i},V_{j} \right)\#\left（ 1 \right） \end{aligned}$$

$$\begin{aligned} d_{J}\left( V_{i},V_{j} \right)=1-J\left( V_{i},V_{j} \right)=1-\frac{\left| V_{i}\cap V_{j} \right|}{\left| V_{i}\cup V_{j} \right|}\#\left（ 2 \right） \end{aligned}$$

where $R$ is the set of regions of one patients in which at least one variation was detected, and $|R|$ is the number of regions in $R$. $V_{i}$ and $V_{j}$indicate the set of variations detected in the $i$st and $j$st region in $R$, respectively. $d_{J}\left( V_{i},V_{j} \right)$ is the Jaccard distance between $V_{i}$ and $V_{j}$, and this distance measures dissimilarity between $V_{i}$ and $V_{j}$ and is complementary to the Jaccard coefficient ($J\left( V_{i},V_{j} \right)$) and obtained by subtracting the Jaccard coefficient from 1. ITHi ranges from 0 (lowest ITH) to 1 (highest ITH). High-ITHi ranges from 0.4 to 1, while low-ITHi ＜ 0.4.

If the tumor has less shared somatic genetic alterations (SNV, indel, gene fusion, CNV) after multi-region sequencing, the ITHi of this tumor will be higher. Otherwise, the ITHi of this tumor will be lower.

**Statistical analysis**

Three datasets including sequencing results from three previously reported NSCLC cohorts (the cancer genome atlas (TCGA)-LUAD, TCGA-LUSC and KEYNOTE-001 [Rizvi N, et al. Science, 2015;348:124-128] ) were obtained to analyze the correlation of TMB from whole exome sequencing (WES) and our panel sequencing. Mutations in these cohorts were filtered by the following four criteria: 1) non-silent, 2) total depth ≥30, 3) at least 5 mutated reads, and 4) mutation frequency ≥3%. Pearson correlation analysis was used to test the linear association between WES and our panel sequencing for TMB, as well as among single-region tTMB, multi-region tTMB and bTMB. A Wilcoxon rank sum test (paired) was used to analyze tTMB fold-change among different enrolled region numbers, as well as multi-region tTMB value among different NSCLC subtypes. All statistical analyses were performed with SPSS (v.21.0; STATA, College Station, TX, USA) or GraphPad Prism (v. 6.0; GraphPad Software, La Jolla, CA, USA) software. Statistical significance was defined as a two-sided *P*<0.05.
