# Supplementary material for: The correlations of tumor mutational burden among single-region tissue, multi-region tissues and blood in non-small cell lung cancer
Source: J Immunother Cancer. 2019 Apr 3;7:98. doi: 10.1186/s40425-019-0581-5 (PMC6448263; doi:10.1186/s40425-019-0581-5)
Supplement: Supplementary file 3 — Figure S1. The correlations of TMB value between panel sequencing and WES in published databases. Figure S2. The comparison of multi-region tTMB among different NSCLC subtypes. Figure S3. The comparisons and overlaps of tumor-derived mutational profiles among tumor tissues in each region and the corresponding ctDNA. (PDF 1439 kb) [file 40425_2019_581_MOESM3_ESM.pdf]

Figure S1. The correlations of TMB value between panel sequencing and WES in published databases.

(**A**) TCGA-LUAD, (**B**) TCGA-LUSC, (**C)** Database from Rizvi H, et al. J Clin Oncol, 2018;36.

Abbreviations: TMB, tumor mutational burden; TCGA, The Cancer Genome Atlas; LUAD, lung adenocarcinoma; LUSC, lung squamous cell carcinoma; WES, wholeexome sequencing

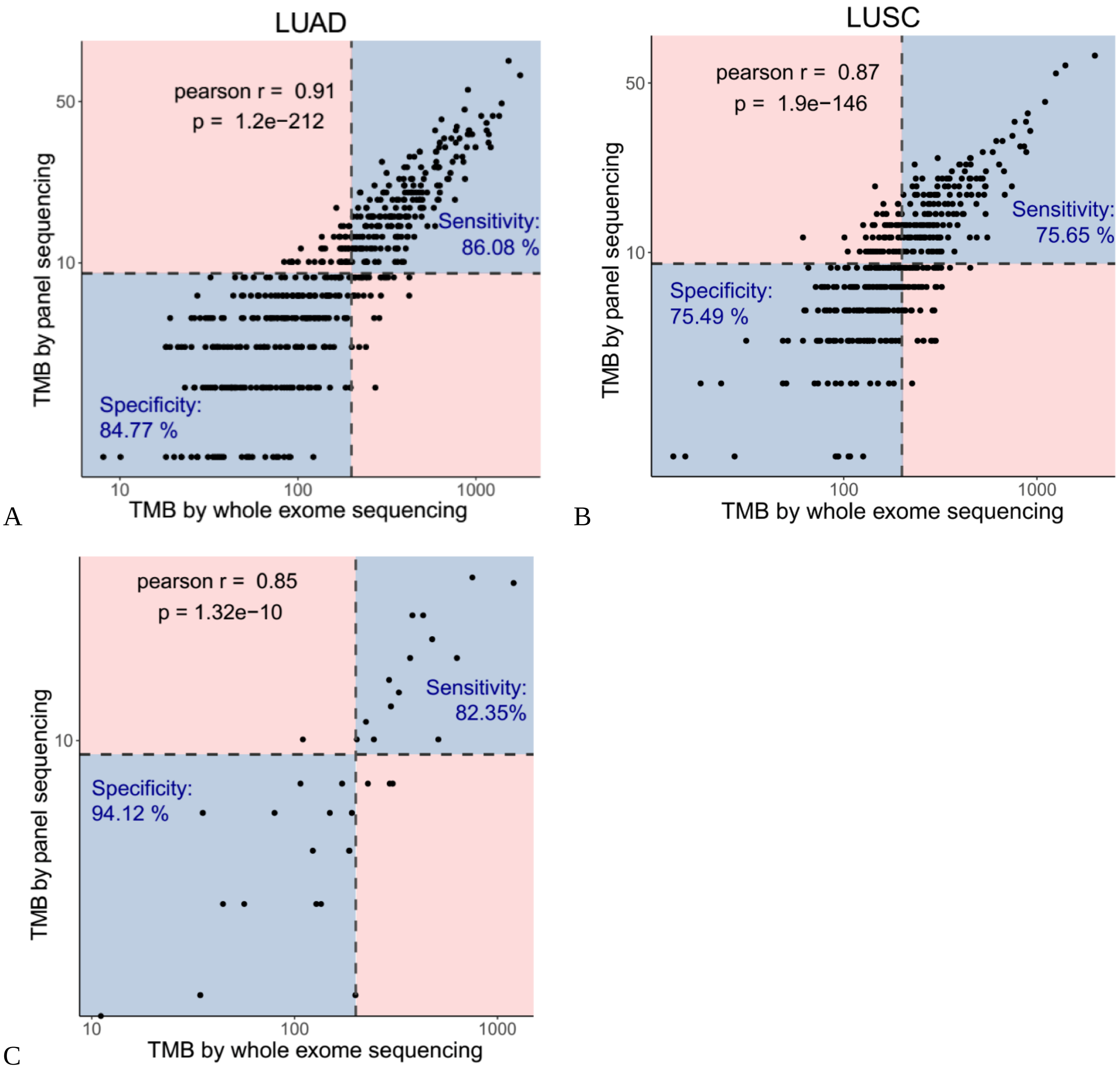

Figure S2. The comparison of multi-region tTMB among different NSCLC subtypes.

Abbreviations: TMB, tumor mutational burden; tTMB, tissue TMB

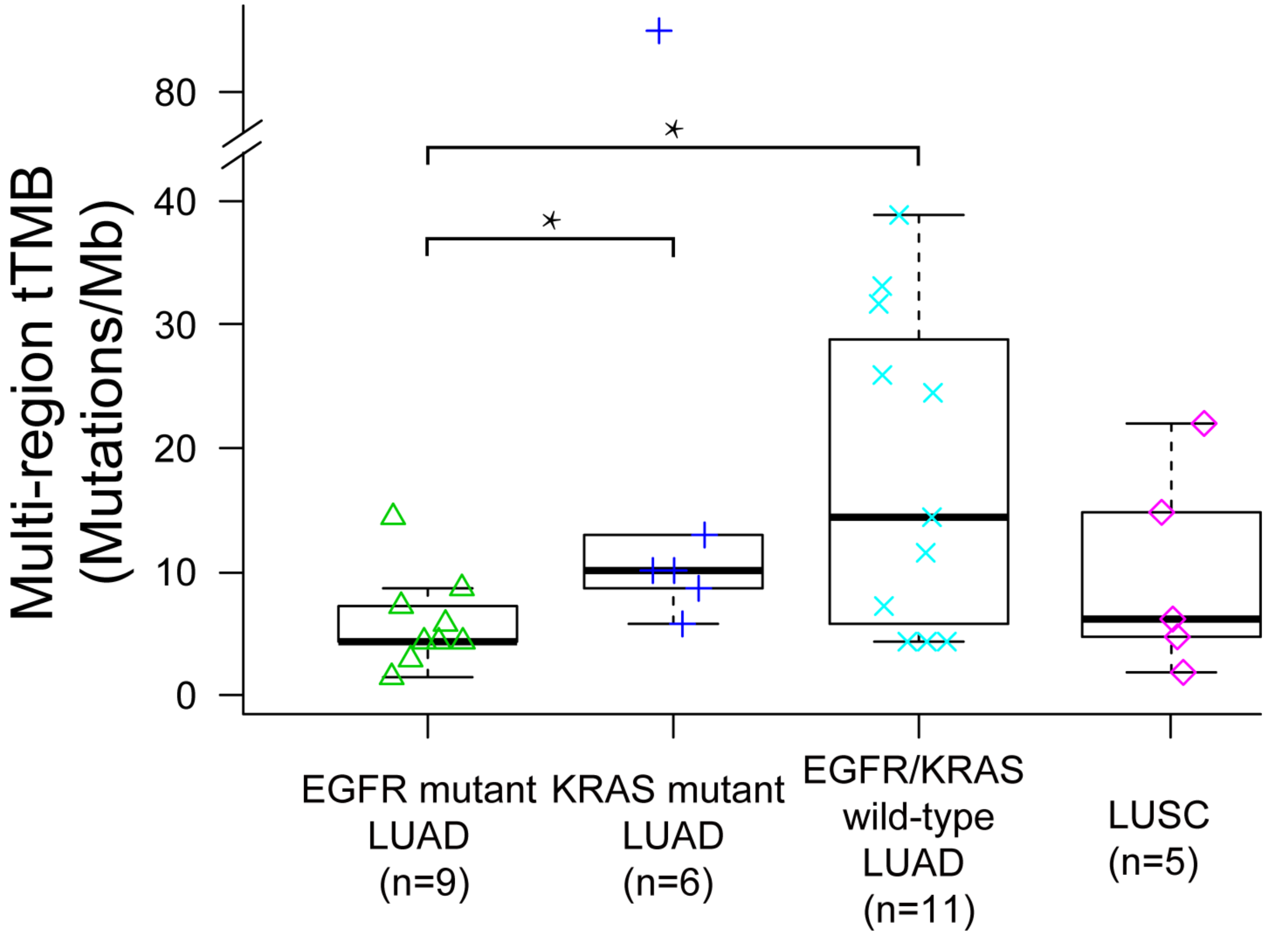

Figure S3. The comparisons and overlaps of tumor-derived mutational profiles among tumor tissues in each region and the corresponding ctDNA.

The P0XX was the patient No. shown at the top. Each tumor region (T1, T2, T3…) with plasma (P) were arranged in the x axis. ctDNA was isolated from plasma (more details in Supplementary Methods). Right y axis displayed tumor-derived mutational profiles in detail. The detected mutations were shown in red, while undetected cases were shown in gray.

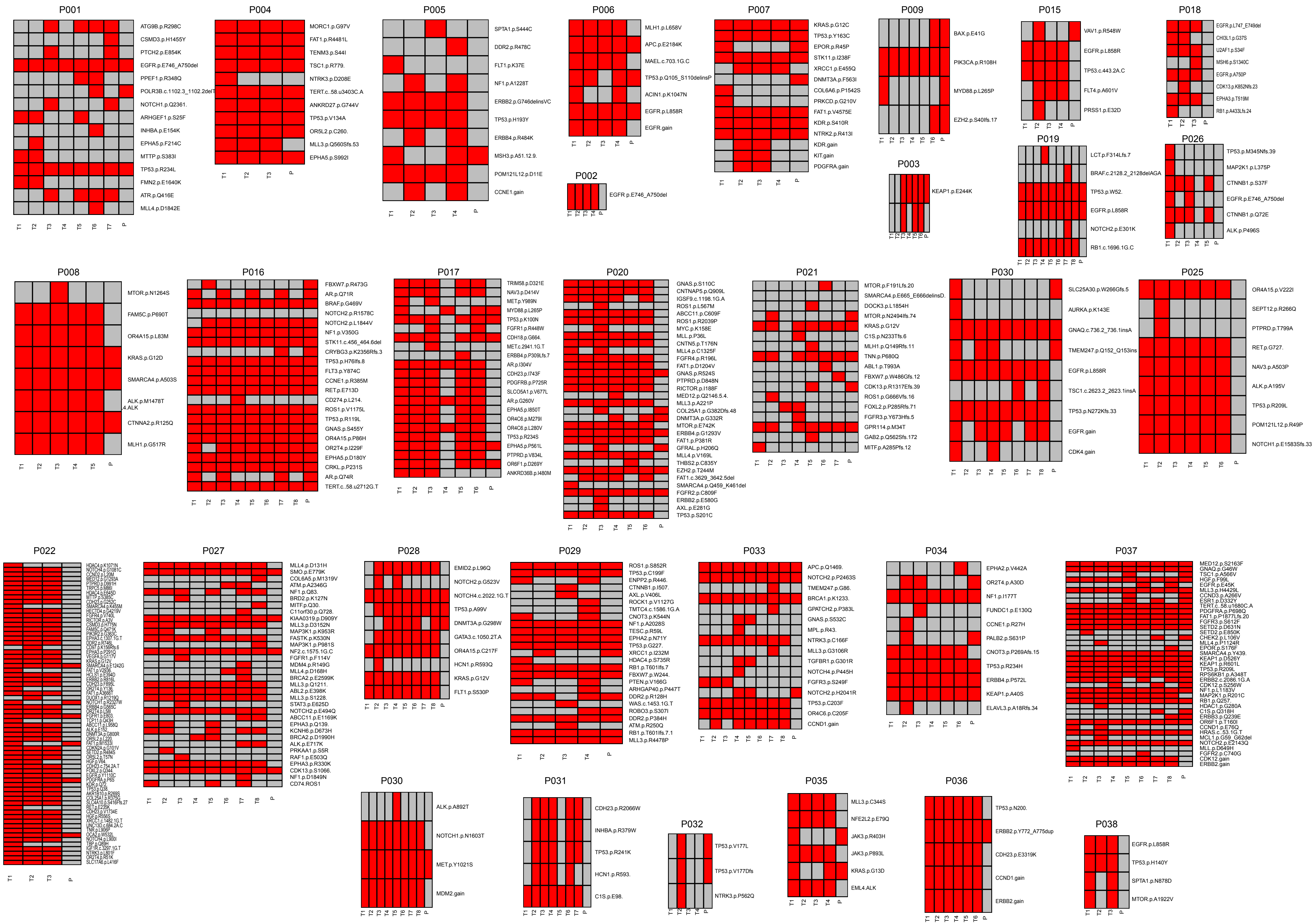
P001
ATG9B.p.R298C
CSMD3.p.H1455Y
PTCH2.p.E854K
EGFR.p.E746_A750del
PPEF1.p.R348Q
POLR3B.c.1102.3_1102.2delT
NOTCH1.p.Q2361.
ARHGEF1.p.S25F
INHBA.p.E154K
EPHA5.p.F214C
MTTP.p.S383I
TP53.p.R234L
FMN2.p.E1640K
ATR.p.Q416E
MLL4.p.D1842E
T1 T2 T3 T4 T5 T6 T7 P
P004
MORC1.p.G97V
FAT1.p.R4481L
TENM3.p.S44I
TSC1.p.R779.
NTRK3.p.D208E
TERT.c..58.u3403C.A
ANKRD27.p.G744V
TP53.p.V134A
OR5L2.p.C260.
MLL3.p.Q560Sfs.53
EPHA5.p.S992I
T1 T2 T3 P
P005
SPTA1.p.S444C
DDR2.p.R478C
FLT1.p.K37E
NF1.p.A1228T
ERBB2.p.G746delinsVC
TP53.p.H193Y
ERBB4.p.R484K
MSH3.p.A51.12.9.
POM121L12.p.D11E
CCNE1.gain
T1 T2 T3 T4 P
P006
MLH1.p.L658V
APC.p.E2184K
MAEL.c.703.1G.C
TP53.p.Q105_S110delinsP
ACIN1.p.K1047N
EGFR.p.L858R
EGFR.gain
T1 T2 T3 T4 P
P002
EGFR p.E746_A750del
T1 T2 T3 T4 P
P007
KRAS.p.G12C
TP53.p.Y163C
EPOR.p.R45P
STK11.p.I238F
XRCC1.p.E455Q
DNMT3A.p.F563I
COL6A6.p.P1542S
PRKCD.p.G210V
FAT1.p.V4575E
KDR.p.S410R
NTRK2.p.R413I
KDR.gain
KIT.gain
PDGFRA.gain
T1 T2 T3 T4 P
P009
BAX.p.E41G
PIK3CA.p.R108H
MYD88.p.L265P
EZH2.p.S40Ifs.17
T1 T2 T3 T4 T5 T6 P
P003
KEAP1.p.E244K
T1 T2 T3 T4 T5 T6 P
P015
VAV1.p.R548W
EGFR.p.L858R
TP53.c.443.2A.C
FLT4.p.A601V
PRSS1.p.E32D
T1 T2 T3 T4 P
P019
LCT.p.F314Lfs.7
BRAF.c.2128.2_2128delAGA
TP53.p.W52.
EGFR.p.L858R
NOTCH2.p.E301K
RB1.c.1696.1G.C
T1 T2 T3 T4 T5 T6 T7 T8 P
P018
EGFR.p.L747_E749del
CHI3L1.p.G37S
U2AF1.p.S34F
MSH6.p.S1340C
EGFR.p.A750P
CDK13.p.K852Nfs.23
EPHA3.p.T519M
RB1.p.A433Lfs.24
T1 T2 T3 P
P026
TP53.p.M345Nfs.39
MAP2K1.p.L375P
CTNNB1.p.S37F
EGFR.p.E746_A750del
CTNNB1.p.Q72E
ALK.p.P496S
T1 T2 T3 T4 T5 P
P008
MTOR.p.N1264S
FAM5C.p.P690T
OR4A15.p.L83M
KRAS.p.G12D
SMARCA4.p.A503S
ALK.p.M1478T
.4.ALK
CTNNA2.p.R125Q
MLH1.p.G517R
T1 T2 T3 T4 T5 P
P016
FBXW7.p.R473G
AR.p.Q71R
BRAF.p.G469V
NOTCH2.p.R1578C
NOTCH2.p.L1844V
NF1.p.V350G
STK11.c.456_464.6del
CRYBG3.p.K2356Rfs.3
TP53.p.H76Ifs.8
FLT3.p.Y874C
CCNE1.p.R385M
RET.p.E713D
CD274.p.L214.
ROS1.p.V1175L
TP53.p.R119L
GNAS.p.S455Y
OR4A15.p.P86H
OR2T4.p.I229F
EPHA5.p.D180Y
CRKL.p.P231S
AR.p.Q74R
TERT.c..58.u2712G.T
T1 T2 T3 T4 T5 T6 T7 T8 P
P017
TRIM58.p.D321E
NAV3.p.D414V
MET.p.Y989N
MYD88.p.L265P
TP53.p.K100N
FGFR1.p.R448W
CDH18.p.G664.
MET.c.2941.1G.T
ERBB4.p.P309Lfs.7
AR.p.I304V
CDH23.p.I743F
PDGFRB.p.P725R
SLCO5A1.p.V677L
AR.p.G260V
EPHA5.p.I850T
OR4C6.p.M279I
OR4C6.p.L280V
TP53.p.R234S
EPHA5.p.P561L
PTPRD.p.V834L
OR6F1.p.D269Y
ANKRD36B.p.I480M
T1 T2 T3 T4 T5 T6 P
P020
GNAS.p.S110C
CNTNAP5.p.Q909L
IGSF9.c.1198.1G.A
ROS1.p.L567M
ABCC11.p.G609F
ROS1.p.R2039P
MYC.p.K158E
MLL.p.P36L
CNTN5.p.T176N
MLL4.p.C1325F
FGFR4.p.R196L
FAT1.p.D1204V
GNAS.p.R524S
PTPRD.p.D848N
RICTOR.p.I188F
MED12.p.Q2146.5.4.
MLL3.p.A221P
COL25A1.p.G382Dfs.48
DNMT3A.p.G332R
MTOR.p.E742K
ERBB4.p.G1293V
FAT1.p.P381R
GFRAL.p.H206Q
MLL4.p.V169L
THBS2.p.C835Y
EZH2.p.T244M
FAT1.c.3629_3642.5del
SMARCA4.p.Q459_K461del
FGFR2.p.C809F
ERBB2.p.E580G
AXL.p.E281G
TP53.p.S201C
T1 T2 T3 T4 T5 T6 P
P021
MTOR.p.F191Lfs.20
SMARCA4.p.E665_E666delinsD.
DOCK3.p.L1854H
MTOR.p.N2494Ifs.74
KRAS.p.G12V
C1S.p.N233Tfs.6
MLH1.p.Q149Rfs.11
TNN.p.P680Q
ABL1.p.T993A
FBXW7.p.W486Gfs.12
CDK13.p.R1317Efs.39
ROS1.p.G666Vfs.16
FOXL2.p.P285Rfs.71
FGFR3.p.Y673Hfs.5
GPR114.p.M34T
GAB2.p.Q562Sfs.172
MITF.p.A285Pfs.12
T1 T2 T3 T4 T5 T6 T7 P
P030
SLC25A30.p.W266Gfs.5
AURKA.p.K143E
GNAQ.c.736.2_736.1insA
TMEM247.p.Q152_Q153ins
EGFR.p.L858R
TSC1.c.2623.2_2623.1insA
TP53.p.N272Kfs.33
EGFR.gain
CDK4.gain
T1 T2 T3 T4 T5 T6 T7 T8 P
P025
OR4A15.p.V222I
SEPT12.p.R266Q
PTPRD.p.T799A
RET.p.G727.
NAV3.p.A503P
ALK.p.A195V
TP53.p.R209L
POM121L12.p.R49P
NOTCH1.p.E1583Sfs.33
T1 T2 T3 T4 T5 T6 P
P022
T1 T2 T3 P
P027
MLL4.p.D131H
SMO.p.E779K
COL6A5.p.M1319V
ATM.p.A2346G
NF1.p.Q83.
BRD2.p.K127N
MITF.p.Q30.
C11orf30.p.Q728.
KIAA0319.p.D909Y
MLL3.p.D3152N
MAP3K1.p.K953R
FASTK.p.K530N
MAP3K1.p.P981S
NF2.c.1575.1G.C
FGFR1.p.F114V
MDM4.p.R149G
MLL4.p.D168H
BRCA2.p.E2599K
MLL3.p.Q1211.
ABL2.p.E398K
MLL3.p.S1228.
STAT3.p.E625D
NOTCH2.p.E494Q
ABCC11.p.E1169K
EPHA3.p.Q139.
KCNH6.p.D673H
BRCA2.p.D1990H
ALK.p.E717K
PRKAA1.p.S5R
RAF1.p.E503Q
EPHA3.p.R330K
CDK13.p.S1066.
NF1.p.D1849N
CD74.ROS1
T1 T2 T3 T4 T5 T6 T7 T8 P
P028
EMID2.p.L96Q
NOTCH2.p.G523V
NOTCH4.c.2022.1G.T
TP53.p.A99V
DNMT3A.p.G298W
GATA3.c.1050.2T.A
OR4A15.p.C217F
HCN1.p.R593Q
KRAS.p.G12V
FLT1.p.S530P
T1 T2 T3 T4 T5 T6 T7 T8 P
P029
ROS1.p.S852R
TP53.p.C199F
ENPP2.p.R446.
CTNNB1.p.I507.
AXL.p.V406L
ROCK1.p.V1127G
TMTC4.c.1586.1G.A
CNOT3.p.K544N
NF1.p.A2028S
TESC.p.R59L
EPHA2.p.N71Y
TP53.p.G227.
XRCC1.p.I232M
HDAC4.p.S735R
RB1.p.T601Ifs.7
FBXW7.p.W244.
PTEN.p.V166G
ARHGAP40.p.P447T
DDR2.p.R128H
WAS.c.1453.1G.T
ROBO3.p.S307I
DDR2.p.P384H
ATM.p.R250Q
RB1.p.T601Ifs.7.1
MLL3.p.R4478P
T1 T2 T3 T4 P
P033
APC.p.Q1469.
NOTCH2.p.P2463S
TMEM247.p.G86.
BRCA1.p.K1233.
GPATCH2.p.P383L
GNAS.p.S532C
MPL.p.R43.
NTRK3.p.C166F
MLL3.p.G3106R
TGFBR1.p.G301R
NOTCH4.p.P445H
FGFR3.p.S249F
NOTCH2.p.H2041R
TP53.p.C203F
OR4C6.p.C205F
CCND1.gain
T1 T2 T3 T4 T5 T6 T7 T8 P
P034
EPHA2.p.V442A
OR2T4.p.A30D
NF1.p.I177T
FUNDC1.p.E130Q
CCNE1.p.R27H
PALB2.p.S631P
CNOT3.p.P269Afs.15
TP53.p.R234H
ERBB4.p.P572L
KEAP1.p.A40S
ELAVL3.p.A18Rfs.34
T1 T2 T3 T4 T5 T6 P
P037
T1 T2 T3 T4 T5 T6 T7 T8 P
P030
ALK.p.A892T
NOTCH1.p.N1603T
MET.p.Y1021S
MDM2.gain
T1 T2 T3 T4 T5 T6 T7 T8 P
P031
CDH23.p.R2066W
INHBA.p.R379W
TP53.p.R241K
HCN1.p.R593.
C1S.p.E98.
T1 T2 T3 T4 T5 T6 T7 P
P032
TP53.p.V177L
TP53.p.V177Dfs
NTRK3.p.P562Q
T1 T2 T3 T4 P
P035
MLL3.p.C344S
NFE2L2.p.E79Q
JAK3.p.R403H
JAK3.p.P893L
KRAS.p.G13D
EML4.ALK
T1 T2 T3 T4 P
P036
TP53.p.N200.
ERBB2.p.Y772_A775dup
CDH23.p.E3319K
CCND1.gain
ERBB2.gain
T1 T2 T3 T4 T5 T6 P
P038
EGFR.p.L858R
TP53.p.H140Y
SPTA1.p.N878D
MTOR.p.A1922V
T1 T2 T3 P
